# Counterproductive Effect of Saccadic Suppression during Attention Shifts

**DOI:** 10.1371/journal.pone.0086633

**Published:** 2014-01-23

**Authors:** Alexandre Zénon, Brian D. Corneil, Andrea Alamia, Nabil Filali-Sadouk, Etienne Olivier

**Affiliations:** 1 Institute of Neuroscience, University of Louvain, Brussels, Belgium; 2 Departments of Physiology & Pharmacology, Psychology, Western University, London, Ontario, Canada; 3 Robarts Research Institute, London, Ontario, Canada; University of California, Davis, United States of America

## Abstract

During saccadic eye movements, the processing of visual information is transiently interrupted by a mechanism known as “saccadic suppression” [1] that is thought to ensure perceptual stability [2]. If, as proposed in the premotor theory of attention [3], covert shifts of attention rely on sub-threshold recruitment of oculomotor circuits, then saccadic suppression should also occur during covert shifts. In order to test this prediction, we designed two experiments in which participants had to orient towards a cued letter, with or without saccades. We analyzed the time course of letter identification score in an “attention” task performed without saccades, using the saccadic latencies measured in the “saccade” task as a marker of covert saccadic preparation. Visual conditions were identical in all tasks. In the “attention” task, we found a drop in perceptual performance around the predicted onset time of saccades that were never performed. Importantly, this decrease in letter identification score cannot be explained by any known mechanism aligned on cue onset such as inhibition of return, masking, or microsaccades. These results show that attentional allocation triggers the same suppression mechanisms as during saccades, which is relevant during eye movements but detrimental in the context of covert orienting.

## Introduction

Visual exploration is performed mostly by moving the eyes and head in order to place the image of objects of interest onto the fovea, the most sensitive portion of the retina. But in addition to this overt exploration mechanism, covert visual attention allows the selection of relevant items in the visual environment, without moving the eyes [4]. The influential premotor theory of attention proposed, more than 30 years ago, that the implementation of attentional shifts results from the covert programming of a saccade which is not executed [3]. In agreement with this view, the same brain structures have been shown to be responsible for overt and covert exploration mechanisms [5]–[7]. However, divergence occurs in these anatomical circuits at the level of neuronal subpopulations [8], [9] and the significance of these distinct circuits for behavior remains an area of active investigation [10]–[16].

During saccadic eye movements, the image projected onto the retina is shifted, inducing a blurring, or retinal slip. We are unaware of this motion because the perception of the movement-induced translation of the visual image is actively suppressed by our visual system, allowing the maintenance of perceptual stability [17]. This mechanism depends on a transient disruption of visual processing around the saccade onset [1], called “saccadic suppression” and is commonly thought to rely, at least partly [18], on an efference copy signal originating from oculomotor centers [2], [19].

If covert attentional shifts are actually implemented through the sub-threshold activation of saccadic control circuits, and if the same circuits are also responsible for the production of the efference copy signal that leads to saccadic suppression, then saccadic suppression may accompany covert attentional shifts. Evidence for this would constitute strong evidence for the functional intertwining of circuits mediating saccades and covert attention, and would suggest that the propagation of efference copy signals can be divorced from actual saccade execution. We attempted to test this prediction in the present study.

**Figure 1:**
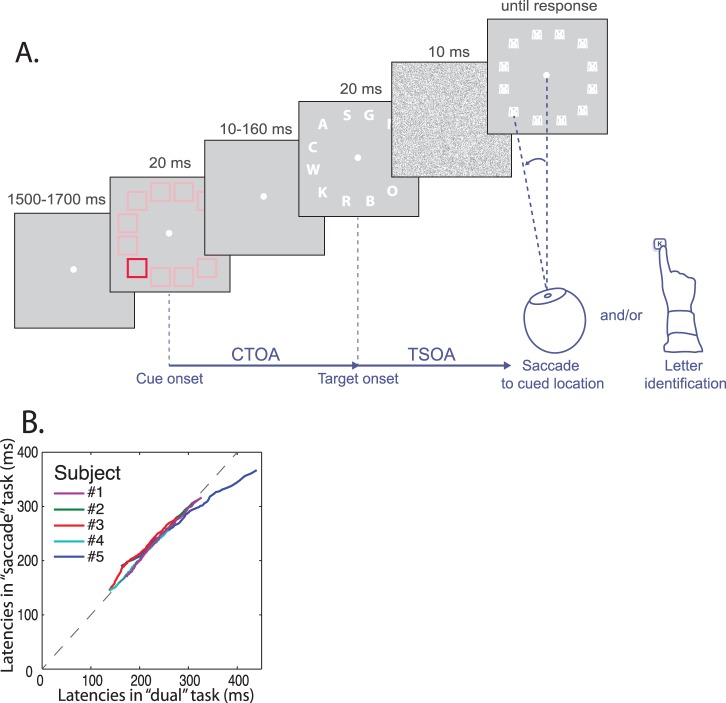
Task used in Experiment 1. A. Schematic depiction of the task. The delay between the cue onset and the target letter onset is called the Cue-Target Onset Asynchrony (CTOA), whereas the delay between letter onset and saccade onset is called the Target-Saccade Onset Asynchrony (TSOA). TSOA equals the CTOA minus the saccade latency and is therefore highly correlated with CTOA. B. Saccadic latencies in the “saccade” task versus the “dual” task: the 1 to 100 latency percentiles in the “saccade” task are plotted against the 1 to 100 latency percentiles in the “dual” task. All subjects but subject n°5 are close to the equivalency line (gray dotted line). In addition, subject n°5 had longer latencies than other subjects.

We asked each of the participants to perform a cued letter discrimination task (the “attention” task), a cued saccade task (the “saccade” task) and a “dual” task including both the saccade and letter discrimination tasks (see Figure 1). Participants were strongly encouraged to orient attention and/or gaze to the cued location, without waiting for the letter display. In each of these three versions of the same task, performed in different blocks, the visual stimulation was strictly identical.

Our approach relied on the following assumption: if covert saccadic preparation occurs during the “attention” task, it will follow the same time course as the one observed in the “saccade” task. If this assumption is true, saccadic latencies measured in the “saccade” task could be used as a marker of the time course of saccadic preparation occurring in the “attention” task. Measuring performance relative to the course of this covert saccadic preparation would then allow us to reveal saccadic suppression. The results of the present study, obtained in two different experiments, provide evidence consistent with this assumption, and demonstrate that saccadic suppression persists in the absence of overt saccade execution.

## Experiment 1

### Materials and Methods

#### Procedure

The present study has been conducted in accordance with the Declaration of Helsinki and has been approved by the local ethics committee (“Commission d’éthique biomédicale hospitalo-facultaire de l’UCL”). Subjects provided their written informed consent prior to participating in the study, following a procedure sanctioned by the ethics committee. Each participant (n = 5, 3 females, ages 19–24) performed three different versions of the same task: the “attention”, the “saccade” and the “dual” tasks. The visual stimulation conditions were identical in the three versions, the only difference being the responses given by the subject. In the “attention” version, participants had to report the identity of a cued letter by typing it on a computer keyboard. In the “saccade” task, they had to perform a saccade to the cued location as quickly as possible. In the “dual” task condition, they had to perform a saccade and identify the cued letter in each trial. In both the “saccade” and “dual” tasks, participants were specifically instructed to orient their gaze to the cue as quickly as possible and not to wait for the display of the target letter.

Each task version was performed in blocks of 40 trials repeated 3–5 times in each session and the order of these blocks was pseudo-randomized. Each of the participants performed 5 or 6 sessions on different days, leading to an average of 3119±417 (mean±SD) trials per subject. At the beginning of each session, a staircase procedure was used to adjust the luminance of the letters in order to reach a performance of 60% in the “attention” task ([20], and see below).

Participants were seated in front of a 19” CRT screen, running at 100 Hz, at a distance of 58 cm, with their head sitting on a chin rest, in a dimly lighted room. Their eye movements were recorded by means of an Eyelink© eye tracker placed below the screen [21]. Their arms were placed on pillows in order to maintain a comfortable position when typing on the computer keyboard.

#### Stimuli

Our task is shown in Fig. 1A. The task began with a 1500–1700 ms display of a fixation point at the center of the screen. The background luminance was at 2% of the maximal luminance of the screen. The fixation point was followed by the display of the cue for 20 ms. The cue was composed of 12 squares 1° wide located on a virtual circle at an eccentricity of 6° from the fixation point. The target square, used to indicate the target location, was always red (RGB: [100%, 2%, 2%]) whereas all the other squares were either reddish ([24%, 2%, 2%]) or of the same color as the background ([2%, 2%, 2%]). The first condition, corresponding to the low salience condition, led to a relatively low contrast of the target square with respect to the other squares, whereas the target square was very conspicuous in the second condition, corresponding to the high salience condition. In the “saccade” and “dual” task conditions, subjects had to perform a saccade to the target cue as quickly as possible. Following the cue, after a variable delay during which only the fixation point was displayed, letters were displayed for 20 ms at the 12 locations corresponding to the location of the squares displayed during the cue epoch. The letter displayed at the location of the target square was the letter that the participants were asked to report in the “attention” and the “dual” tasks. The delay between the offset of the cue and the onset of the target, or cue-target onset asynchrony (CTOA), was either 10, 40, 80, 120 or 160 ms.

The displayed letters were all different and were chosen randomly in the Latin alphabet, except the letter M and X, which were never used (as told to the participants at the beginning of the experiment). Letters were followed by a series of 2 masks. The first mask, displayed for 10 ms encompassed the whole screen and consisted of Gaussian noise. The second mask consisted of the superimposition of the letters M and X and the number “8”, displayed at each of the 12 locations where letters were displayed and lasted until the subject typed the letter on the keyboard (“attention” and “dual” tasks) or for a maximum of 1000 ms.

The staircase procedure, performed at the beginning of each session, used the same visual stimulation conditions. The CTOA delay was always 120 ms and the salience of the cue was always high. The luminance obtained through this staircase procedure (31±12% of the screen maximal luminance, mean±SD), leading to a performance of the subject in the letter discrimination task of around 60%, was used for the remaining of the session.

#### Microsaccade detection

We identified microsaccades using a similar method as described by [22]. In all subjects, movement amplitude was strongly correlated with movement velocity, confirming the saccadic nature of the eye movements (“main sequence”, all p values <.0001). The amplitude ranged from about.1 to 2 degrees.

#### Analysis

In order to assess the effect of Target-Saccade Onset Asynchrony (TSOA) on performance in the “dual” task, we first sorted all trials in increasing order of TSOA, then binned the trials by groups of 20. We then fitted two different functions on these performance data. One consisted of a logistic-transformed linear regression with 3 parameters a, b and c: and was called the “attention fit” model. The other was the product of 2 such logistic-transformed linear regressions , had 5 parameters a, b, c, d, and e, and was called the “attention and suppression fit” model. The first function is monotonic and was used to model only the effect of attention being progressively allocated to the cue. This function does not model the suppression effect which occurs close to saccade onset. In contrast, the second function is not monotonic and takes into account both the performance increase induced by progressive attentional allocation and the later decrease observed when the letter display was close to saccade onset.

For the “attention” task, TSOA values were estimated from the latencies measured during the “saccade” task of the same session, in the corresponding salience, CTOA and position condition, leading to 4 trials per data point. We then binned the data in the same way as with the “dual” task (20 trials per bin) and fitted the two models described above.

Fitting was performed using a non-linear regression based using least squares estimation (nlinfit in Matlab©). Residuals were systematically inspected, ensuring the normality of their distribution and the absence of any systematic relation with fitted values. Comparison between the two models was performed by computing the Bayes Factor on the basis of the proportion of variance explained [23]. We also compared the “attention and suppression” model fitted on the “attention” performance data as described above with the same analyses conducted on shuffled TSOA data, repeated 1000 times (bootstrap method). This comparison was made by looking at the distribution of the difference in proportion of variance explained between the real and shuffled data. A distribution of such differences significantly larger than zero, as assessed by a Student t-test was considered as a proof that the non-shuffled TSOA data provided more information than the shuffled data, thus allowing to predict better the letter discrimination performance. Analyses were performed both subject-by-subject and on the 4-subject population altogether, then including a subject random factor.

### Results

#### Comparison of the performance in the “attention”, “saccade” and “dual” tasks

In order to be able to use the saccadic latencies measured in the “saccade” task as a potential estimate of the covert saccadic preparation time in the “attention” task, we first had to make sure that these two tasks were performed similarly. In order to demonstrate this, we compared 1) the saccadic latencies in the “dual” and “saccade” tasks and 2) the discrimination performance in the “dual” and “attention” tasks.

We tested each of the 5 subjects separately and found that only one of them showed a significant difference in saccadic latency distribution (log-transformed) between the “attention” and “saccade” tasks, on the one hand, and the “dual” task, on the other hand (subject n°5, see Fig. 1 B). We also tested for each subject whether the effect of the different task conditions on performance differed between the “dual” and the “attention” or “saccade” tasks (3-way ANOVA with Cue-Target Onset Asynchrony or CTOA, salience and task version as independent variables and either letter discrimination rate or log saccade latencies as dependent variables). Here again, only subject n°5 showed a significant interaction between the task factor and the other factors (p<0.05). These idiosyncrasies can be partly explained by the lack of compliance of subject n°5 to the instructions regarding the necessity not to wait for the target appearance before making a saccade. As a consequence, we did not include this subject in the analyses described hereafter in this paper.

The 3-way ANOVA described above allowed us to assess also the effect of CTOA on saccade latencies. We found that, in 2 of the subjects, CTOA condition affected latencies significantly (p<.05), with longer CTOAs leading to longer latencies. However, this was true in both the “dual” and the “saccade” tasks (TASK×CTOA interaction, all p values >.1). The fact that this effect was also present in the “saccade” task indicates that it was not due to the subjects waiting for the letter display to initiate the saccade. Therefore, we expected this effect not to impact on our results in the set of analyses described below.

#### Time course of attentional allocation in the “dual” task

**Figure 2:**
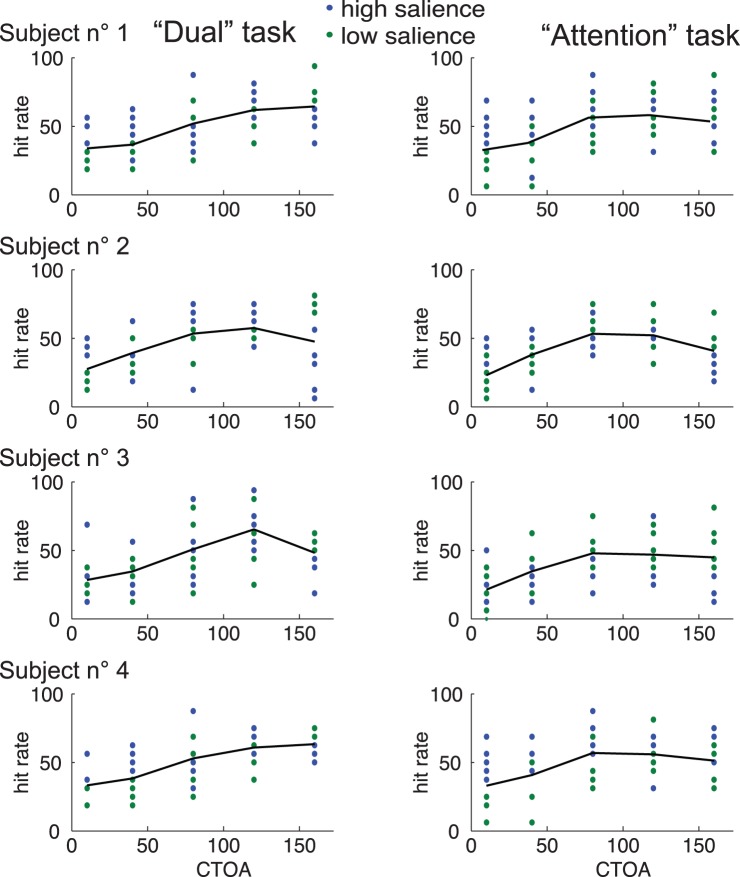
Performance in letter discrimination as a function of CTOA condition for each subject in the “dual” task (left half of the figure) and the “attention” task (right half of the figure). Each dot corresponds to a group of 2 blocks in the low (in green) or high (in blue) salience condition. The black curves indicate the means irrespective of salience condition.

**Figure 3:**
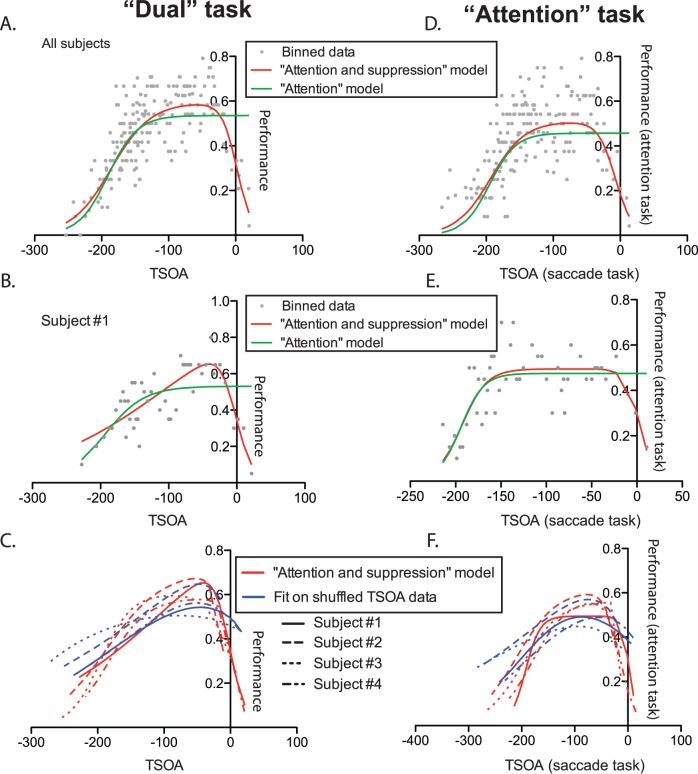
Effect of Target-Saccade Onset Asynchrony (TSOA) on letter discrimination rate. Panels A to C correspond to the “dual” task, whereas panels D to F are for the “attention” task. Saccade onset corresponds to a TSOA value of zero. Each dot corresponds to 20 trials. The curves in green and red show the fits obtained with the “attention” and “attention and suppression” models, respectively. Panels A and D show the data from all the subjects mixed together while panels B and E show the data from subject #1 only and panels C and F show the “Attention and suppression” fits (in red) obtained for each subject as well as the fits obtained on the shuffled data (in blue).

We found that performance was low for early CTOA conditions and increased with larger CTOA values (see Figure 2), indicating that, following cue onset, spatial attention was progressively allocated to the cue. However, we were mostly interested in the time course of attentional allocation as a function of saccade onset. In order to investigate this particular aspect of attentional time course, we looked at the letter discrimination performance as a function of TSOA (see Fig. 1A). These analyses, which were performed on data from the “dual” task requiring both letter detection and an overt saccade, are illustrated in the left half of Fig. 3. The position of each grey data point on the Y-axis indicates the average performance in 20 trials and its position on the abscissa corresponds to the average TSOA for these trials. We compared 2 different models (see Methods) and found that, on the 4-subject population as a whole, the model accounting the best for the data was the one taking account of the performance decrease close to saccade onset (“attention and suppression” model, illustrated in red in Figure 3A, Bayes factors (BF) superior to 10^10^ when compared to the simpler “attention” model, shown in green in Fig. 3D). The parameters corresponding to the increasing and decreasing slopes of this function were both significantly different from zero (see Methods, confidence interval (CI) for parameters *a*: [0.0275 0.1290] and *d:* [−0.0525 −0.0254], alpha = .05). This shows that discrimination performance changed with TSOA in two ways. First, it increased as attention was progressively allocated to the cue, at TSOAs going from low values (corresponding to short CTOAs) to about −100 ms (see Fig. 3A). Second, it decreased as the target letter got displayed closer to the time of saccadic onset, with TSOAs getting closer to zero.

We also performed the same analysis subject by subject. In each subject, we also found that the “attention and suppression” model was favored over the simpler “attention” model. We found very strong evidence in one subject (BF equal to 3314), positive evidence in 2 (BF equal to 6.15 and 14.92) and only weak evidence in 1 (BF equal to 1.7). The fitted curves obtained are shown in red (“attention and suppression” model) and green (“attention” model) in Figure 3B (subject #1).

Because TSOA and CTOA are strongly correlated with each other (TSOA = CTOA-saccadic latency), it is difficult to determine which of the two parameters influences perceptual performance the most. In order to determine whether TSOA was really more informative that CTOA to predict attention time course, we fitted the “attention and suppression” model to the data after having shuffled the saccade latencies. We performed this analysis 1000 times and compared the proportion of variance explained in each of these fits with the proportion of variance explained with the non-shuffled data. In each subject the proportion of variance explained was superior in the non-shuffled data and this difference was highly significant (Wilcoxon signed rank test, all p values <.0001). The average of the 1000 fitted curves obtained on the shuffled data for each subjects is shown in blue in Figure 3C.

#### Saccadic suppression with covert attentional allocation

To test our hypothesis that covert saccade preparation induces saccadic suppression, we analyzed the letter discrimination performance from the “attention” task as a function of saccade latency from the “saccade” task. We reasoned that, since participants performed these tasks similarly to the “dual” task, as demonstrated above, the saccade latencies in the “saccade” task could be used as a marker of saccade preparation time in the letter discrimination task.

Strikingly, we obtained results very similar to those observed in the “dual” task, including a strong decrease in performance for inferred TSOA values close to zero, even though a saccade was never actually executed. Here again, the best fit to the TSOA data was obtained with the “attention and suppression” model (Bayes factors superior to 10^6^ when compared to the “attention” only model, see Figure 3D). The slope parameters of that regression were significantly different from zero (CI for *a:* [0.0275 0.1290], CI for *d:* [−0.0525 −0.0254], alpha = .05).

When performed subject by subject, the same analysis provided strong evidence in favor of the “attention and suppression” model for one subject (BF = 8024), but only weak evidence for the 3 other subjects, one of them showing even a weak preference for the simple “Attention” model (BF = 0.23, see Figure 3E).

In order to ensure that the saccade latency gathered in the “saccade” task really provided information that helped to take into account the variance of the performance, we repeated the same analysis as above but after shuffling the TSOA data within each subject. The fit obtained from the average coefficients of these 1000 non-linear regressions are shown in blue in Figure 3F. A Wilcoxon signed rank test confirmed that for each subject, these distributions differed from zero (p<.0001), confirming that the saccadic latencies obtained in the “saccade” task helped significantly in predicting the performance time course during the “attention” task.

#### Microsaccades cannot account for TSOA-locked changes in performance

Microsaccades are eye movements known to affect perceptual performance and therefore, even though no eye movements larger than 2 degrees occurred during the “attention” task, we had to ensure that microsaccades were not responsible for the drop in performance observed for short TSOAs. Indeed it could be hypothesized that microsaccades happened more often around zero TSOAs because it is the time around which the saccade would be performed if it were allowed.

**Figure 4:**
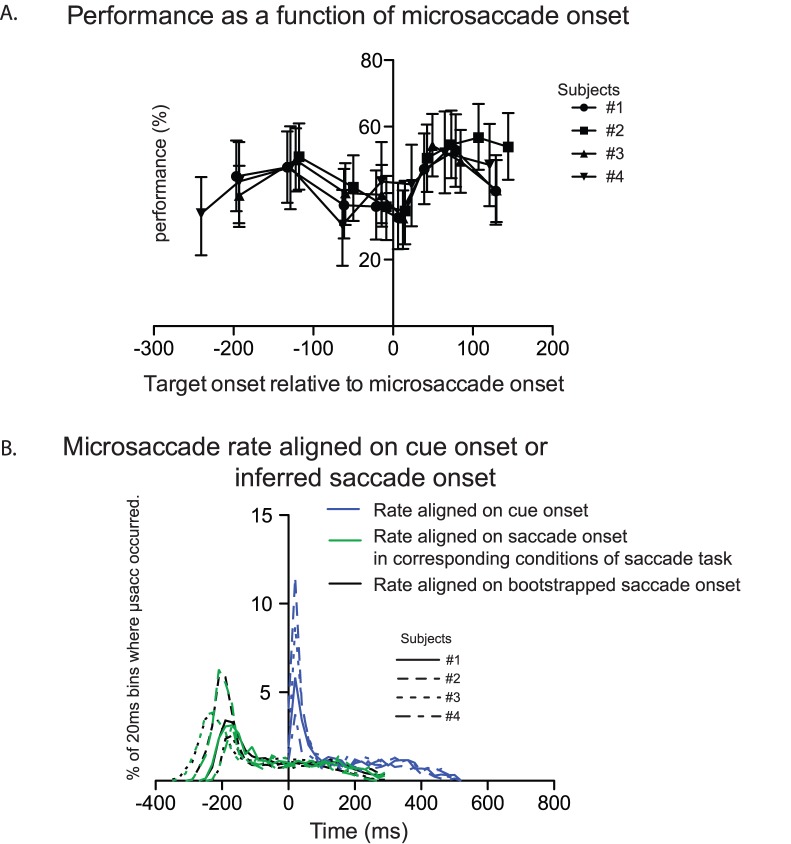
Microsaccade analysis in Experiment 1. A. Performance in letter discrimination as a function of delay with respect to microsaccade onset. Error bars indicate confidence intervals. B. Rate of microsaccade aligned on cue onset (blue), inferred saccade onset (green) or shuffled inferred saccade onset (black).

We first confirmed that microsaccades affected performance in our task. For each subject, we measured performance as a function of target onset relative to microsaccade onset, in 10 equal-size bins (see Figure 4A). Pairwise comparisons showed that in all subjects, performance was lower around the microsaccade onset (between around −70 and +15 ms; all p values <.05) than following microsaccades (between around +60 and +110 ms).

Although performance did decrease around the time of microsaccades, these eye movements were most often time-locked to the cue onset and cannot explain the effect of TSOA on performance detailed above. To show this, we measured the rate of microsaccades in each 20 ms time bin following cue onset. We found for all subjects a very sharp increase in microsaccades following the trial onset (see blue trace in Figure 4B). Similarly to the analysis described above on the effect of TSOA on performance, we aligned the microsaccade rate measured in the “attention” task on the latency of saccades measured in the “saccade” task (green trace in Figure 4B) or on the shuffled latencies (black trace in Figure 4B). The striking similarity of the green and black curves indicates that taking into account saccade latency in the “saccade” task does not add any relevant information and that microsaccade rate is affected by cue onset rather than covert saccadic preparation.

Importantly, these results confirm that the drop in performance observed for TSOAs close to zero cannot be explained by the occurrence of an eye movement before or during the letter display.

#### Effect of TSOA within each CTOA condition

We have described up to now the time course of perceptual performance aligned on saccade onset (actual or inferred). However, as mentioned above, we had to ensure that the effects of TSOA we observed on perceptual performance were not caused by changes in performance depending on the delay with respect to the onset of the cue, i.e. as a function of CTOA (see Fig. 1A). Multiple regressions are sometimes used to disentangle such correlated explanatory variables. However, we didn’t apply this method to our data because the correlations were too high (Variance Inflation Factors ranged between 2.4 and 3.29, [44]), the effects were not linear and the results of a multiple regression would not necessarily help us in confirming or refuting the existence of a saccadic suppression phenomenon.

**Figure 5:**
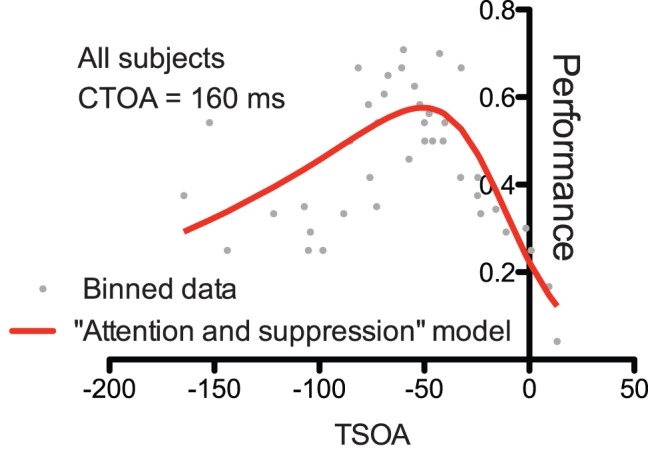
Effect of TSOA on letter discrimination performance within the 160 ms CTOA condition in the “attention” task. This analysis was performed on the data of all subjects together.

Another potential approach to this issue is to consider the fact that, if the decrease of performance for TSOA nearing zero were caused by a stimulus-locked phenomenon, we would not expect to find an effect of TSOA on performance when computed for a single CTOA level. Instead, we found that even in the 160 ms CTOA condition only, the “attention and suppression” model provided a better fit to the data than the “attention only” model (Fig. 5, Bayes Factor = 7.55). This analysis had to be performed on the data integrating all the subjects together since the number of trials in this condition was to small to allow a subject-by-subject analysis. These results, which show an effect of TSOA in a single CTOA condition, demonstrate that the effect of TSOA on performance and, most importantly, the decrease in performance observed when TSOA gets close to zero, cannot be explained by the effect of CTOA alone, or by any mechanism linked to the timing of the stimuli. Instead, the time course of the subject’s performance in the “attention” task requires to take account of the subject’s habitual saccadic latencies, even if saccades are not generated.

### Discussion

In Experiment 1, we have shown that, in a dual saccadic and attentional task, the time course of perceptual performance is time-locked to the preparation and execution of the saccade, in agreement with earlier studies [16], [24]–[27]. Critically, we extended this finding to a covert attention task, by inferring the time course of saccadic preparation from the latencies of saccades performed in an overt version of the same task. In particular, we found that performance drops dramatically when the letter to be discriminated is displayed right before the time at which the saccade would have been executed. We interpret these results as evidence for the existence of saccadic suppression in the covert attention task.

Even though the population analyses provided highly significant results, we failed to find systematically significant effects on a subject-by-subject basis. The main reason is that in some subjects, in too few trials, the TSOAs fell in the relevant range, close to zero ms. In order to tackle this issue, we conducted a second experiment in which we changed the CTOA values (70, 110, 150, 190, 230 and 270 ms) in order to have a larger proportion of trials with TSOAs appropriate to unveil the saccadic suppression effect.

In addition, it could be argued that the use of the masks in Experiment 1, consisting of overlaid letters “X” and “M”, could be problematic, because saccadic suppression could affect the perception of the masks themselves, and disrupt the performance time course in an unpredictable way. Therefore, we decided to discontinue using these masks in Experiment 2, and used instead individual static circular masks, consisting of random noise filtered at 3 cycles/letter [28], and which stayed on the screen during the whole task duration.

## Experiment 2

### Materials and Methods

**Figure 6:**
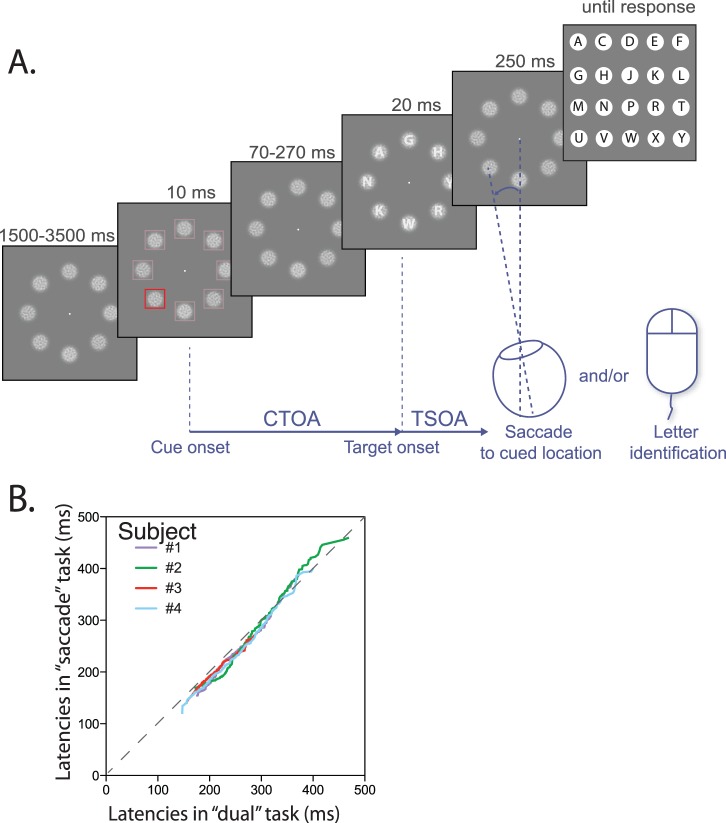
Task used in Experiment 2. A. Schematic depiction of the task. B. Saccadic latencies in the “saccade” task versus the “dual” task in Experiment 1. Same convention as in Figure 1.

In Experiment 2, we used the same task as in Experiment 1, except for the following details (see Fig. 6A): 4 subjects participated, all different from Experiment 1, consisting of 3 females, aged 28.5±4.65; CTOA values were either 70, 110, 150, 190, 230 or 270 ms; in each letter location, and during the whole task duration, a 3°-wide mask was displayed, consisting of noise filtered at 3 cycles/letter [28]; letters were displayed as an increase in luminance over this mask and the amount of luminance increase was determined during a staircase procedure prior to each session, similarly to Experiment 1; only 8 masks and 8 letters were displayed in each trial instead of 12; the letters B, I, O, Q, S and Z were not used [29]; after each trial, a keypad was shown on the screen and the subject had to respond by clicking on the corresponding letter. Blocks of “saccade”, “attention” and “dual” tasks were alternated in each session, in pseudo-random order. One session consisted thus in 3 blocks. In each block there was one trial of each condition (6 CTOA levels, 4 positions, 2 salience levels), leading to a total of 48 trials per block. The rest of the procedure was identical to Experiment 1.

The analysis was also performed similarly to Experiment 1, by first sorting the data in increasing order of TSOA, then binning the data by groups of 24 trials and finally fitting a nonlinear function on the binned data. For the covert attention analysis, we used, for each trial, the latency obtained in the “saccade” version of the task, and the performance in letter detection in the “attention” task of the same session, in the corresponding condition. Similarly to Experiment 1, we compared the goodness of fit of two different functions: “attention fit”, identical to Experiment 1, and “attention and suppression” fit. This latter function differed from Experiment 1 in that it included an additional quadratic term to take account of the performance recovery observed with large positive TSOAs, following the saccadic suppression: . Because the large range of CTOAs led to an unnecessarily wide range of TSOAs, we restricted the analysis to TSOA values between −100 and +100 ms.

### Results

We first checked that the saccade latencies in the “dual” and “saccade” versions of the tasks were similar, and that the effect of the different conditions on performance did not differ between the “attention” and the “dual task”, following the same procedure as in Experiment 1 (see Fig. 6B, all p values >.1).

**Figure 7:**
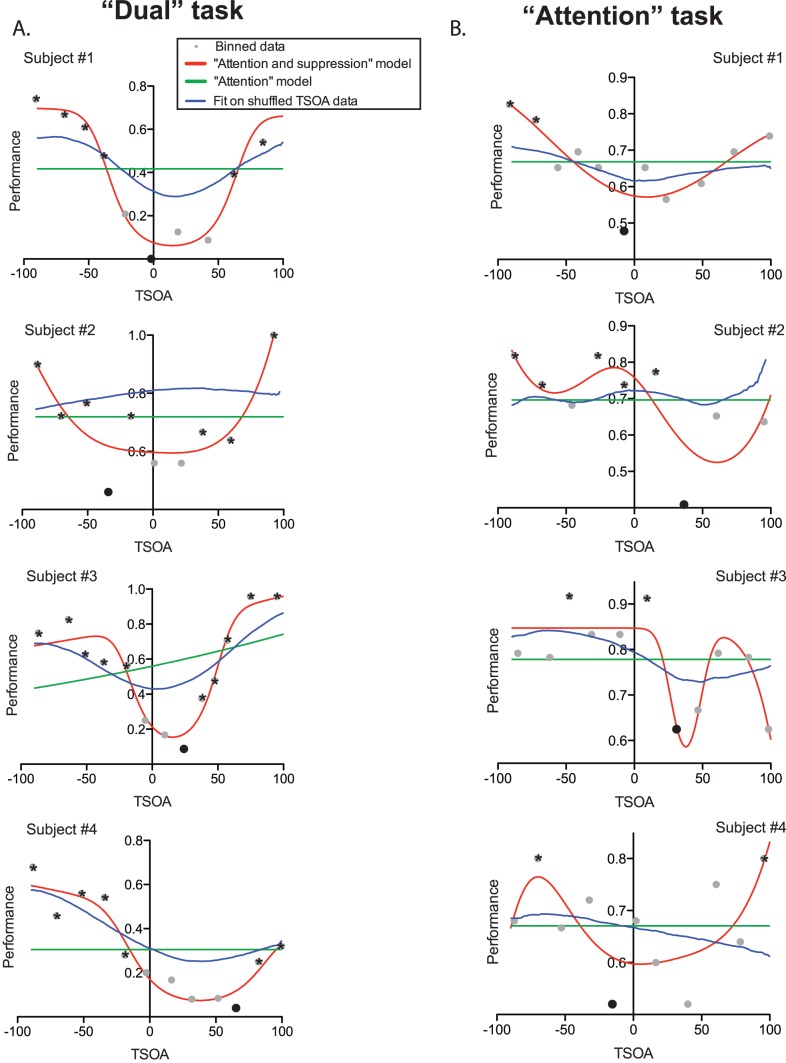
Performance in letter discrimination as a function of TSOA for the “dual” (A) and the “attention” (B) tasks, for each subject. The green curves show the fit obtained with the “attention” model, whereas the red and blue curves show the fit obtained with the “attention and suppression” model on the actual and shuffled data, respectively. Each point corresponds to 24 trials. The bin associated to the poorest performance is shown in black and the bins significantly different from it are marked with an asterisk.

We then analyzed the time course of performance variation as a function of TSOA, subject by subject. In the “dual” version of the task, we found that the “attention and suppression” function fitted the data significantly better than the “attention only” fit (Bayes Factor >10^6^, except for subject 3 for whom BF = 22); this confirms the results from Experiment 1, showing that saccadic suppression occurred during this task (see Fig. 7A, red and green curves respectively). It is noteworthy that, given the large CTOA values used in Experiment 2, the ascending part of the “attention only” model was not covered by the range of TSOA values, leading to flat curves. Importantly, pairwise bins comparison showed that the bins associated to the minimal performance (in black in Fig. 7A, TSOA values: −1.69, −34.19, 24.15, 65.30 for subjects #1 to #4, respectively) were significantly different from at least 2 other bins (all p<.01, Fisher exact test, significant bins shown with the asterisk in Fig. 7A). We also ran the same bootstrap analyses as in Experiment 1, with randomly shuffled saccadic latencies. The percentage of variance explained was significantly higher with the un-shuffled than the shuffled data for all subjects (see Fig. 7A, blue curves, Wilcoxon signed rank test: all p<.0001).

In the “attention” task, in all subjects, comparison of the “attention” and the “attention and suppression” models showed positive evidence in favor of the “attention and suppression” (see Fig. 7B, red and green curves, Bayes Factors: 257, 4.94, 1656, 4.73 for subject #1 to #4, respectively), including, for three out of four subjects, a recovery of performance following the drop aligned on the predicted saccade onset. Pairwise bins comparisons led to the same results, with bins close to zero TSOA being significantly different from at least 2 other bins (see Fig. 7B, black markers and asterisks, Fisher exact test, all p values <.05, TSOA values of minimal bins: −7.46, 36.39, 30.91, −15.39). The comparison of the fits obtained with the actual data and with the shuffled data showed that the percentage of variance explained with the actual data was significantly higher in all subjects (see Fig. 7B, blue curves, Wilcoxon rank sum test: all p<.0001).

**Figure 8:**
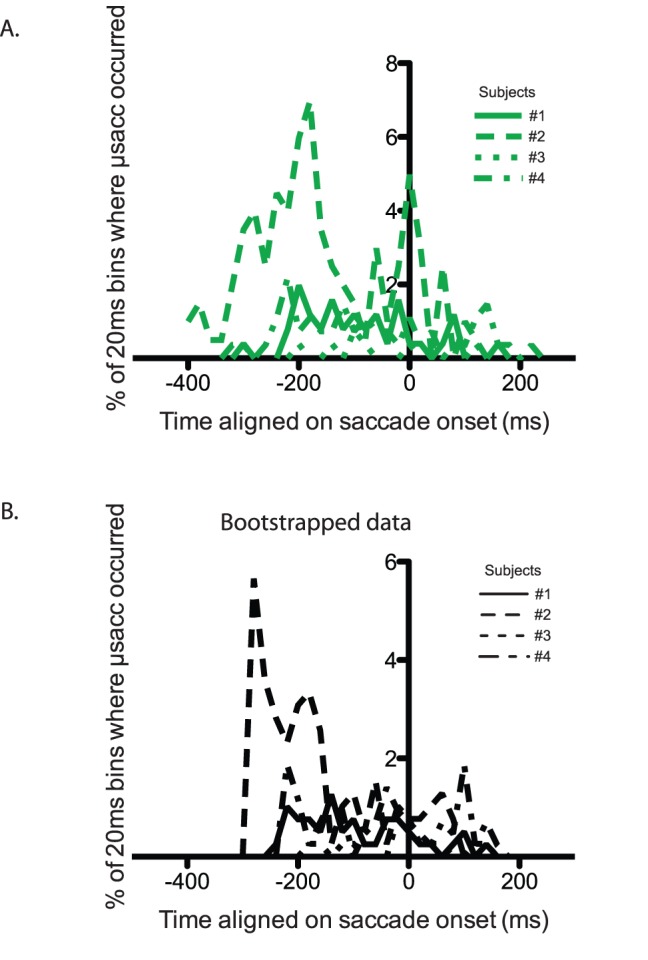
Microsaccade analysis in Experiment 2. A. Rate of microsaccades in the “attention” task as a function of delay with respect to inferred saccade onset. B. Same analysis, performed on shuffled saccadic latencies.

Finally, we also performed the same analysis as in Experiment 1 on the microsaccades. In general, the rate of microsaccades in the “attention” task was very low, and was not aligned on inferred saccade onset (see Fig. 8). However, we found, for subject #2 only, a small peak in microsaccades rate in the bin centered on 0 ms delay. In order to ensure that this small peak could not explain the drop in performance observed around this TSOA value, we looked at all the trials in which these microsaccades occurred (n = 10). In 6 trials only, the letter display followed the microsaccades, and in 4 out of these 6 trials, the response was correct. Thus, this small microsaccade peak at 0 ms TSOA for subject #2 cannot explain the results we observed in the letter discrimination performance.

### Discussion

In Experiment 2, we replicated the findings of Experiment 1 by showing that in both the overt and covert versions of the task, perceptual performance dropped around the actual or inferred saccade onset. Experiment 2 led also to the following additional results. First, the exact timing of the performance drop around the saccade onset varied between subjects, both in the overt and covert conditions. Second, in all subjects, the saccade-aligned performance drop was followed by a strong recovery in the overt condition, because, in these trials, the target letter was projected onto the fovea. Third, in all but one subject, we observed a similar recovery in the covert condition, despite the fact that in this case, the target letter never appears on the fovea. Finally, in all subjects, the magnitude of the saccadic suppression was smaller in the covert than in the overt condition. This was expected given that the analysis of the covert condition relies on saccadic latency data coming from a different task, which can provide only a rough estimation of what the actual timing of saccadic preparation is during the covert trials. This lack of timing precision leads unavoidably to a smoothing of the perceptual time course and to a decrease of the magnitude of saccadic suppression. Therefore, we cannot conclude from this data, that the magnitude of saccadic suppression is smaller in covert than in overt attention shifts.

## General Discussion

In two different experiments, we found that performance in a covert attention task dropped significantly at the end of the inferred saccadic preparation, indicative of the presence of saccadic suppression despite the absence of actual saccade.

In this study, we made the atypical choice of using a letter discrimination task to investigate saccadic suppression. The phenomenon of saccadic suppression is thought to depend mostly on the magnocellular pathway [1]. Even though the stimuli we used were not optimized to probe magnocellular vision, we must emphasize that letter discrimination depends also on low spatial frequencies [28] and that the magnocellular vision network is known to have a role, even possibly prominent, in letter identification [30]. In agreement with this view, saccadic suppression has been shown previously to affect letter identification [29]. Therefore, the use of a letter identification task in the present study was appropriate to study saccadic suppression.

As stated in the Introduction, the approach we used here is based on the strong assumption that the time course of saccadic preparation in the “saccade” task can be used as a marker of saccadic preparation during the “attention” task. We tried to provide evidence for the validity of this assumption by showing that discrimination performance and saccadic latencies were similar when performed alone or in the dual version of the task. But most importantly, our finding that the time course of discrimination performance relative to saccadic preparation remained the same in the overt and the covert versions of the task can be regarded as a strong *a posteriori* validation of this assumption.

Although the present results show that covert selection of a peripherally cued stimulus leads to a phenomenon of saccadic suppression, it remains uncertain whether this finding would generalize to any attentional task. In particular, it is possible that a task involving central, instead of peripheral cueing, or a task placing less emphasis on rapid saccadic or attentional orienting would lead to different results. Indeed, tasks involving central cues have been shown to lead to perceptual improvements around the time of saccadic onset in dual tasks [12], [25], [31]. However, these results were based on data having a lower temporal resolution than the present study, and therefore might have failed to encompass the time window where saccadic suppression occurs. Further work will thus be needed to determine whether saccadic suppression in covert orienting task is specific to peripheral cueing.

The present results have several important implications. First, they show that instead of consisting of a monotonic increase of perceptual performance, attentional allocation leads to a more complex time course, with an initial increase followed by a later decrease in performance. Crucially, this performance decrease depends on the timing of the saccade preparation and not simply on the delay following cue presentation. Therefore, all possible alternative explanations of our results relying solely on stimulus-locked phenomena, such as inhibition of return [32], the time course of the sustained component of attention [33], or visual masking [34], [35] can be ruled out. In fact, it is possible that some of these stimulus-locked phenomena may be partly caused by the saccadic suppression mechanisms observed in the present study. Testing this intriguing possibility would require different experiments involving non-informative cues in addition to informative ones, longer delays following cue onset and the assessment of reaction times in addition to accuracy.

A second significant consequence of the present results is that they show that saccadic suppression does not depend on processes related to the execution of the saccade, but are already triggered during saccadic preparation. This is especially relevant in the context of another ongoing debate regarding the role of backward masking in saccadic suppression [18], [36]–[38], [43]. The observation of a comparable effect of saccadic suppression in the presence or absence of actual saccade execution shows that, at least in the present task, the efference copy signal is the most significant drive of the saccadic suppression and that backward masking plays only a minor role.

Finally, the third important implication of the present study is with regard to the interaction of the brain circuits involved in the control of eye movements and covert attention. Whereas the existence of an overlap between these brain structures is no longer questioned [11], most of the debate is now focused on the stage at which these circuits diverge at the level of neuron subpopulations and on the precise role these circuits play in behavior [8], [9], [13], [14], [39], [40]. The present results imply that the neurons involved in relaying the efference copy signal of the saccadic program [19] are also activated during covert attentional shifts. This is especially interesting because, in this particular case, their activation leads to a disruption of visual performance that is counterproductive in the context of covert orienting. This further suggests that covert attentional allocation relies on the recruitment of a neural circuit for which eye movement control, and not covert selection, is the primary function. Interesting candidates are the subcortical circuits involving the Superior Colliculus (SC). Recent findings have shown that the SC, in addition to its well-known function in eye movement control, plays a causal role in visual selection, independently of the attention-related modulations observed in visual cortex [41], [42]. The SC is also thought to relay the efference copy signals involved in saccadic suppression, through its pulvino-cortical connections [2]. The present results suggest that visual selection and saccadic suppression mechanisms engage a common set of neurons within the SC-pulvino-cortical circuit, and that their activation during attentional allocation could send automatic saccadic suppression signals to visual cortex.
